# Supplementary material for: Politics overwhelms science in the Covid-19 pandemic: Evidence from the whole coverage of the Italian quality newspapers
Source: PLoS One. 2021 May 20;16(5):e0252034. doi: 10.1371/journal.pone.0252034 (PMC8136646; doi:10.1371/journal.pone.0252034)
Supplement: S1 File — (DOCX) [file pone.0252034.s001.docx]

**Supporting Information for:** **“Politics overwhelm science in the Covid-19 pandemic: Evidence from Italian quality newspaper coverage”**

**S1. Datasets**

The overall dataset is organized around three nested corpora. The first corpus (i.e. “total corpus”) includes all the articles published in the timespan between January 1, 2020 and June 15, 2020. The second corpus (i.e. “SARS-CoV-2 general corpus”) is a subset of the “total corpus”, as it includes only articles containing at least one of the following terms: [covid, corona virus, OR coronavirus]. The third corpus (i.e. “SARS-CoV-2 focused corpus”, see S1 Tab) derived from the previous one through the selection of articles related to the thematic domains emerging from the topic modelling (see section 4 of this document). The newspaper articles published in the timespan between January 1–June 15, 2020 have been collected by means of a dedicated media monitoring platform developed by the research group [Details withheld to preserve blind review]. None of the records have been duplicated. Articles with less than 50 characters were excluded because they were mainly short photo-gallery or video descriptions. Data acquisition relied on online news such as RSS feeds associated with specific newspaper sections obtained through a collector module. This media monitoring platform allows the user to collect, archive, and index daily newspaper articles from several countries. The indexing module provides efficient access to the articles via metadata and content-based searches using Information Retrieval technologies [1]. Currently, the platform monitors quality press in different languages and countries: the eight most important Italian newspapers (*Il Corriere della Sera*, *La Repubblica*, *La Stampa*, *Il Sole – 24 Ore*, *Avvenire*, *Il Giornale*, *Il Messaggero*, and *Il Mattino*), five news organisations in English (*The New York Times*, *The Guardian*, *The Mirror*, *The Telegraph*, and *The Times of India*), six in French (*Le Figaro*, *Lacroix*, *Le Monde*, *Les Echos*, *Liberation*, and *Parisien*), the 100 most relevant Italian blogs, and approximately 100 Twitter accounts in Italian [4]. The current analysis relied on the Italian newspapers, each of which contributed to the general corpus as illustrated in S1 Tab.

**S1 Table. SARS-CoV-2 general corpus distribution across newspapers (n = 54,477).**

| **Newspaper Title** | **Contribution to the SARS-CoV-2 general corpus (%)** | **Orientation** |
| --- | --- | --- |
| *Corriere della Sera* | 11.82% | Moderate |
| *La Repubblica* | 12.68% | Progressive |
| *Il Sole* – *24 Ore* | 6.42% | Neoliberal |
| *La Stampa* | 30.19% | Moderate |
| *Avvenire* | 4.23% | Catholic |
| *Il Giornale* | 11.47% | Conservative |
| *Il Mattino di Napoli* | 10.86% | Moderate |
| *Il Messaggero* | 12.33% | Moderate |

**S2. Periodization**

The analysis relied on custom periodization based on the series of breaking events that punctuated the entire first semester of 2020. This enabled the authors to give an account of how the emergency developed in Italy, as well as to inform the interpretation process of the topics produced through LDA (see Section 4 of Supporting Information). The periods and their main features (i.e., starting and ending date, breaking events, articles published) are listed in S2 Tab.

**S2 Table. Periods considered, their main features and SARS-CoV-2 general corpus (%) distribution across periods (n = 54,477).**

| **Period** | **Start** | **End** | **Breaking Events** | **Contribution to SARS-CoV-2 general corpus (%)** |
| --- | --- | --- | --- | --- |
| 1 | Jan 1, 2020 | Jan 31, 2020 | – First cases officially registered in Italy  – Closure of flights from China to Italy | 1.06% |
| 2 | Feb 1, 2020 | Feb 22, 2020 | – First COVID-19 death officially recorded in Italy  – First Prime Ministerial Decree, schools in Veneto and Lombardy closed | 3.28% |
| 3 | Feb 23, 2020 | March 1, 2020 | – Prime Ministerial Decree March 1, 2020 | 4.82% |
| 4 | March 2, 2020 | March 8, 2020 | – Schools and universities closed in the whole country  – Prime Ministerial Decree March 8, 2020 | 5.26% |
| 5 | March 9, 2020 | March 16, 2020 | – Beginning of the lockdown  – Professional sporting events cancelled  – New decree for public health funding | 8.73% |
| 6 | March 17, 2020 | March 21, 2020 | – First extension of the lockdown  – Closure of non-essential productive activities | 6.27% |
| 7 | March 22, 2020 | April 1, 2020 | – Closure of parks all over the country  – Further restrictions to individual mobility | 12.88% |
| 8 | April 2, 2020 | April 10, 2020 | – Second and third extension of the lockdown | 9.92% |
| 9 | April 11, 2020 | April 26, 2020 | – Prime Ministerial Decree April 26 announcement of Phase 2 | 14.26% |
| 10 | April 27, 2020 | May 4, 2020 | – Re-opening of non-essential productive activities | 6.54% |
| 11 | May 5, 2020 | May 13, 2020 | – New economic measures for a “Relaunch Package” | 7.04% |
| 12 | May 14, 2020 | May 18, 2020 | – Further re-opening for commercial activities (e.g., clothing stores) | 3.27% |
| 13 | May 19, 2020 | May 26, 2020 | – Prime Ministerial Decree May 26: Re-opening | 5.51% |
| 14 | May 27, 2020 | June 3, 2020 | – Mobility across the country allowed | 4.52% |
| 15 | June 4, 2020 | June 15, 2020 | – Professional sporting activities re-open | 6.63% |

**S3. Classifier**

With the aim to analyze all of the newspaper articles in the reporting period we tested different machine-learning (ML) techniques to identify which articles had relevant scientific content. Then, the scientific salience (i.e., the ratio of these articles to the total number of articles published in a given time span) was measured for both the “total corpus” and the “SARS-CoV-2 general corpus”.

Initial candidate classification algorithms were chosen: Random Forest, Naïve Bayes, Nearest Neighbor, Multinomial Naïve Bayes (MNB), Linear Stochastic Gradient Descent (LSGD), Dual Coordinate Descent method for Logistic Regression (DCD-LR), and Support Vector Machine (Least Squares Support Vector Machine – LS-SVM, and divide-and-conquer solver for kernel SVMs – DC-SVM). Their implementation was available in JSAT, an ML library for the Java language. For each of these ML algorithms, three different measurements of the results’ accuracy were considered: precision, recall, and F_1_-score. The procedure involved a five-fold cross-validation for a selected hyperparameter on a sample of 3,814 articles in Italian that were appropriately labelled. Then, the algorithms with an F_1_-score and a recall (in order to minimize the number of false positives) above 0.80 were selected. These algorithms were Multinomial Naïve Bayes, Linear Stochastic Gradient Descent, and Logistic Regression DCD. For each of these, a five-fold cross-validation was used to select the best combination of values for the models’ hyperparameters using the same algorithm (S3 Tab. 1). The assessment of the five-fold cross validation was done on a training set including 80% of the documents in the sample (3,051 of 3,814 documents). In all cases, the articles are represented in the form of *v_d_* numerical vectors, where *v_d_[i]* is the number of times (term frequency) that the index term *i* appears in the *d* document.

**S3 Table 1. Hyperparameter cross-validation results for three models.**

| **MULTINOMIAL NAÏVE BAYES (MNB)** | | | | |
| --- | --- | --- | --- | --- |
| Hyperparameter: *smoothing* | | | | |
| **Hyperparameter** | **Precision** | **Recall** | **F_1_** | **Error Rate** |
| **0.001** | 0.882 | 0.87 | 0.88 | 0.075 |
| **0.01** | 0.861 | 0.907 | **0.88** | 0.073 |
| **0.1** | 0.835 | 0.925 | 0.88 | 0.078 |
| **1** | 0.835 | 0.915 | 0.87 | 0.08 |
|  |  |  |  |  |
| **LINEAR STOCHASTIC GRADIENT DESCENT (LSGD)** | | | | |
| Hyperparameter: *regularization L1* (loss function: HingeLoss) | | | | |
| **Hyperparameter** | **Precision** | **Recall** | **F_1_** | **Error Rate** |
| **0.0001** | 0.931 | 0.827 | **0.88** | 0.072 |
| **0.001** | 0.926 | 0.825 | 0.87 | 0.073 |
| **0.01** | 0.925 | 0.781 | 0.85 | 0.087 |
| **0.1** | 0.912 | 0.624 | 0.74 | 0.133 |
|  |  |  |  |  |
| **LOGISTIC REGRESSION DUAL COORDINATE DESCENT (DCD-LR)** | | | | |
| Hyperparameter: *regularization* (number of iterations was also tested; no significant results) | | | | |
| **Hyperparameter** | **Precision** | **Recall** | **F_1_** | **Error Rate** |
| **0.0001** | 0.951 | 0.626 | 0.76 | 0.124 |
| **0.001** | 0.916 | 0.836 | 0.87 | 0.073 |
| **0.01** | 0.909 | 0.854 | **0.88** | 0.071 |
| **0.1** | 0.9 | 0.848 | 0.87 | 0.075 |

Once the optimal values of the hyperparameters for the three models were found, their generalizability was compared to the test set, obtained from the remaining 763 articles (20%) of the sample. In this comparison, three other models were tested:

(1) a custom classifier based on the multiplicative weights of some keywords (Keyword M)

(2 & 3) two classifiers exploiting the stacking functionality of JSAT by combining and weighting the predictions of the selected classifiers. The first one (Stacking 3) combines the classifications of MNB, LR-DCD, and LSGD; the second one (Stacking 2) combines the classifications of LR-DCD and LSGD

**S3 Table 2. Final comparison of the algorithms.**

| **Algorithm** | **Precision** | **Recall** | **F_1_** | **Error Rate** |
| --- | --- | --- | --- | --- |
| **Keyword M** | 0.82 | 0.95 | 0.88 | 7.73% |
| **MNB** | 0.84 | 0.93 | 0.88 | 7.60% |
| **DCD-LR** | 0.89 | 0.9 | 0.9 | 6.85% |
| **LSGD** | 0.9 | 0.87 | 0.88 | 6.94% |
| **Stacking 3** | 0.88 | 0.93 | 0.91 | 6.02% |
| **Stacking 2** | 0.91 | 0.88 | 0.9 | 5.70% |

Of all the classifiers, the one that provided the greatest accuracy with the lowest overall error rate and highest F_1_-score was the Stacking 2 classifier (combining Logistic Regression DCD and Linear Stochastic Gradient Descent). In this way, it was possible to determine whether the content of each article pertained to the scientific domain (with an error rate <6%) and to calculate the relative weight of science in all the published articles (salience) in the selected time span.

**S4. LDA**

LDA is a generative probabilistic model for unsupervised classification that treats each document as a mixture of topics and each topic as a mixture of words. LDA aims to reveal the hidden topics and each document’s distribution over topics. Topics are represented by multinomial distributions over a vocabulary consisting of words from all documents in a large-scale corpus. The topic modeling generative process assumes that each document is created by first being assigned to a distribution over topics; then, each word in a document is selected from a topic according to the document’s distribution over topics.

Corpus pre-processing included tokenization (word unit identification), discarding punctuation, word capitalization (all capital letters were converted to lowercase), and filtering out stop-words (functional words such as prepositions, articles, etc.). Even when working through the bag-of-words approach, the main multi-word expressions were analyzed by tokenizing adjacent words into n-grams and simultaneously visualizing recurrent relationships among words (Figure A1). In some cases, n-grams were recoded into unigrams or acronyms to monitor whether they appeared among the top words of the topics [6]. This procedure enabled careful detection of certain personalities (politicians, scientists, etc.) and organizations (WHO, ECB^[[1]](#footnote-1)^, etc.) and assess their relevance in the topics without ambiguity. Lemmatization was avoided because in large datasets, lemmatizing words can be harmful because it ignores information in the conjugated forms [3].

**S4 Fig. The Most frequent bigrams.**


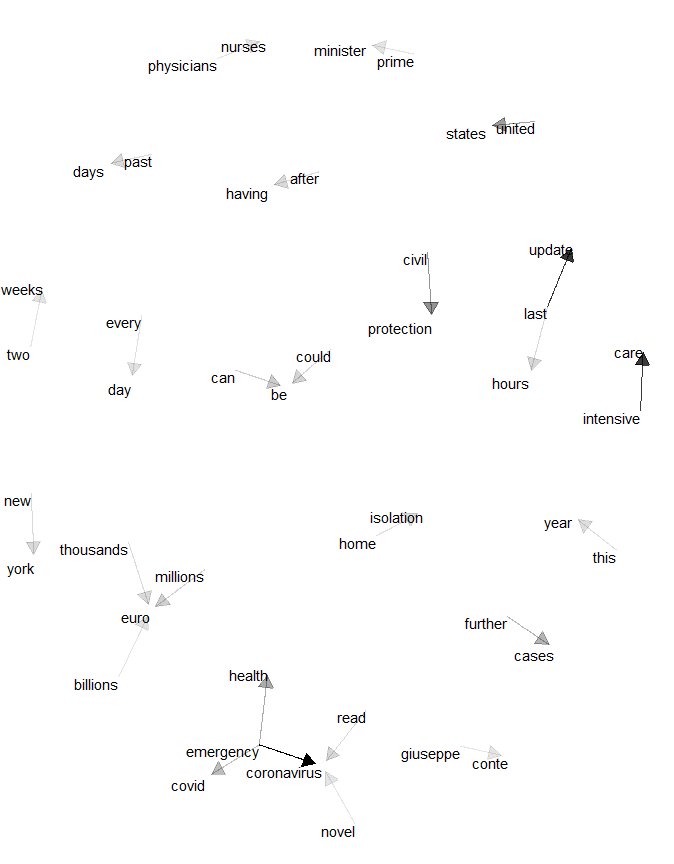


The initial corpus had 397,964 types and 14,043,971 tokens (type/token ratio: 2.8%). After pre-processing and stop-word removal, the corpus had 216,827 types and 10,608,034 tokens (type/token ratio: 2.0%). Therefore, the corpus was sufficiently extensive for statistical analysis, even though the thresholds reported in the literature are only broad indications and can often be distorted depending on the context or the extent of the corpus [5].

The implementation was available in Mallet, a Java-based package for statistical natural language processing, document classification, and topic modeling.

The initial values of hyperparameters were set to

K = 50

α = 5/K (Mallet default)

β = 0.01 (Mallet default)

Here, K represents the number of topics, α represents the document-topic density, and β represents the word-topic density. In this way, it was expected that the documents would contain few topics, and that the topics would be made up of few words. Of course, the initial values can change after hyperparameter optimization deployed via Mallet.

By varying the number of topics, several candidate models were run and compared for significant differences, interpretability, and avoiding overlapping between topics [2]. In the first run, 50 topics were extracted from the dataset. All the topic descriptions, consisting of the top words per topic, were then manually scrutinized to select topics pertaining to the Sars-CoV-2 pandemic. The selected topics included three major components:

(1) explicit reference to issues of healthcare, disease, and illness related to the Sars-CoV-2 pandemic

(2) explicit reference to healthcare and biomedical agencies and public policies for managing the Sars-CoV-2 pandemic

(3) explicit reference to biomedical research and medical technologies to address the Sars-CoV-2 pandemic

Sars-CoV-2-related content played a marginal role in most of the excluded topics, which were mainly connected to other issues such as sport or economic and financial news. Hence, the datasets were refined to only include articles for which one of the selected topics was most relevant (in terms of topic proportion). In this way, through the first LDA run, the number of articles constituting the datasets was reduced from 58,646 to 54,477. This more specific dataset was analyzed through a second LDA run. In this second run, the number of topics for extraction was set to 40 to obtain more specific topics (i.e., boosting their sensitivity to grasp particular and well-bounded issues relevant for addressing the research questions). The most pertinent topics were detected following the same approach adopted in the first run and using the same three inclusion criteria. Subsequently, with the removal of three irrelevant topics (concerning local news or articles related to art and literature), the final number of topics (i.e., 37) was determined by a theoretically motivated choice and obtained through a data-driven approach: in this way, the “general corpus” was constructed.

A qualitative investigation performed within this “general corpus” allowed the identification of three main thematic domains: politics, science, and medicine. Consensual decision confirmed which topics would be included in the three thematic domains; here, the 13 most pertinent were selected. In this way, a “focused corpus” was built consisting of 15,487 articles in which the pandemic was related to the three thematic domains mentioned above.

Then, 913 terms were selected from the 13 topics assigned to the thematic frames (100 top words for each topic) by assessing their relevance in terms of the probability of being generated from a selected topic. The decision to recode some of the multi-word expressions and proper nouns into unigrams was useful, as most of these appeared among the top words. Subsequently, the top words were classified into the categories of organizations, people, and positions and in the areas of medicine, politics, science, and technique. Overall, 82 terms were classified into these two dimensions (S4 Tab). Finally, the frequencies of this subset of terms was analyzed in the sub-corpus comprising articles that, according to the LDA posterior probabilities, showed a dominant feature of the three thematic frames.

**S4 Table. Frequencies of the selected terms (sub-corpus of politics, science, and medicine).**

| **Area** | **Category** | **Term** | **Frequency** |
| --- | --- | --- | --- |
| Technique | People | Angelo_Borrelli | 1,220 |
| Technique | People | Domenico_Arcuri | 974 |
| Technique | Organizations | civil_protection | 2,596 |
| Technique | Organizations | ECB | 1,761 |
| Technique | Organizations | INPS | 1,662 |
| Technique | Organizations | ISTAT | 597 |
| Science | Roles | specialist | 2,880 |
| Science | Roles | researchers | 1,710 |
| Science | Roles | professor | 1,671 |
| Science | Roles | scientists | 1,158 |
| Science | Roles | virologist | 823 |
| Science | People | Silvio_Brusaferro | 1,039 |
| Science | People | Burioni | 467 |
| Science | Organizations | università (ITA) | 2,858 |
| Science | Organizations | WHO | 2,561 |
| Science | Organizations | labs | 943 |
| Science | Organizations | university (EN) | 609 |
| Science | Organizations | AIFA | 513 |
| Politics | Roles | mayor | 2,463 |
| Politics | Roles | minister | 2,312 |
| Politics | Roles | prime_minister | 1,993 |
| Politics | Roles | majority | 1,973 |
| Politics | Roles | governor | 1,507 |
| Politics | Roles | leader | 1,395 |
| Politics | Roles | opposition | 1,228 |
| Politics | Roles | assessor | 1,184 |
| Politics | Roles | centre-right | 762 |
| Politics | Roles | members_of_parliament | 651 |
| Politics | Roles | deputy | 432 |
| Politics | Roles | group_leader | 299 |
| Politics | Roles | undersecretary | 283 |
| Politics | Roles | senator | 253 |
| Politics | People | Giuseppe_Conte | 6,927 |
| Politics | People | Matteo_Salvini | 2,502 |
| Politics | People | Roberto_Gualtieri | 1,582 |
| Politics | People | Attilio_Fontana | 1,531 |
| Politics | People | Roberto_Speranza | 1,377 |
| Politics | People | Silvio_Berlusconi | 1,230 |
| Politics | People | Giuseppe_Gallera | 1,141 |
| Politics | People | Giogia_Meloni | 1,099 |
| Politics | People | Ursula_von_Der_Leyen | 1,012 |
| Politics | People | Sergio_Mattarella | 926 |
| Politics | People | Luca_Zaia | 913 |
| Politics | People | Renzi | 823 |
| Politics | People | Luigi_Di_Maio | 790 |
| Politics | People | Alfonso_Bonafede | 678 |
| Politics | People | Vincenzo_De_Luca | 649 |
| Politics | People | Merkel | 625 |
| Politics | People | Francesco_Boccia | 537 |
| Politics | People | Zingaretti | 522 |
| Politics | People | Lagarde | 521 |
| Politics | People | Giuseppe_Sala | 479 |
| Politics | People | Giovanni_Toti | 268 |
| Politics | Organizations | parliament | 1,756 |
| Politics | Organizations | ministry | 1,626 |
| Politics | Organizations | health_ministry | 1,441 |
| Politics | Organizations | chamber_of_deputies | 1,409 |
| Politics | Organizations | executive_branch | 1,182 |
| Politics | Organizations | governments | 1,156 |
| Politics | Organizations | palazzo_chigi | 962 |
| Politics | Organizations | European_Union | 943 |
| Politics | Organizations | Eurogroup | 868 |
| Politics | Organizations | senate | 841 |
| Politics | Organizations | interior_ministry | 733 |
| Politics | Organizations | parties | 637 |
| Politics | Organizations | Lega | 452 |
| Politics | Organizations | FdI | 353 |
| Medicine | Roles | patients | 10,636 |
| Medicine | Roles | physicians | 8,529 |
| Medicine | Roles | health_workers | 2,168 |
| Medicine | Roles | nurses | 1,973 |
| Medicine | Roles | general_practitioners | 869 |
| Medicine | Roles | physicians_&_nurses | 652 |
| Medicine | Roles | head_physician | 614 |
| Medicine | Roles | veterinarian | 155 |
| Medicine | Organizations | hospital | 10,989 |
| Medicine | Organizations | RSA | 2,586 |
| Medicine | Organizations | ISS | 2,187 |
| Medicine | Organizations | ASL | 2,126 |
| Medicine | Organizations | emergency_department | 1,303 |
| Medicine | Organizations | general_hospital | 854 |
| Medicine | Organizations | pharmacy | 314 |

**References**

1. Di Buccio E, Lorenzet A, Melucci M, Neresini F. Unveiling Latent States Behind Social Indicators. In Proceedings of the First Workshop on Data Science for Social Good co-located with European Conference on Machine Learning and Principles & Practice of Knowledge Discovery in Databases, SoGood@ECML-PKDD 2016, Riva del Garda, Italy, September 19, 2016. (R. Gavaldà, I. Zliobaite, and J. Gama, eds.), vol. 1831 of CEUR Workshop Proceedings, CEUR-WS.org

2. Maier D, Waldherr A, Miltner P, Wiedemann G, Niekler A, Keinert A, Pfetsch B, Heyer G, Reber U, Häussler T, Schmid-Petri H. Applying LDA topic modeling in communication research: Toward a valid and reliable methodology. CommMet and Meas. 2018 Apr 3;12(2-3):93-118.

3. Matsumoto S, Takamura H, Okumura M. Sentiment classification using word sub-sequences and dependency sub-trees. InPacific-Asia conference on knowledge discovery and data mining 2005 May 18 (pp. 301-311). Springer, Berlin, Heidelberg.

4. Neresini F. Old media and new opportunities for a computational social science on PCST. Journal of Science Communication. 2017 Jun 21;16(2):C03.

5. Vermeer A. Coming to grips with lexical richness in spontaneous speech data. Lan test. 2000 Jan;17(1):65-83.

6. Wallach HM. Topic modeling: beyond bag-of-words. InProceedings of the 23rd international conference on Machine learning 2006 Jun 25 (pp. 977-984).

1. European Central Bank. [↑](#footnote-ref-1)
