# Supplementary material for: Politics overwhelms science in the Covid-19 pandemic: Evidence from the whole coverage of the Italian quality newspapers
Source: PLoS One. 2021 May 20;16(5):e0252034. doi: 10.1371/journal.pone.0252034 (PMC8136646; doi:10.1371/journal.pone.0252034)
Supplement: Attachment — Submitted filename: response_to_reviewers.pdf [file pone.0252034.s002.pdf]

APRIL 2021

Dear Reviewers and Assigned Academic Editor,

Thank you for the opportunity to revise and resubmit the manuscript "Politics overwhelms science in the Covid-19 pandemic: evidence from the whole coverage of the Italian quality newspapers" to be considered for publication as a research article in PLOS ONE.

The reviewers' suggestions were highly insightful and enabled us to improve the quality of our manuscript since they identified conceptual and methodological issues that needed clarification.

We believe we have found a solution that addresses all the main issues and concerns raised by the reviewers. Accordingly, we have integrated the required revisions, and we highlighted the changes to our manuscript within the document by using colored text.

Below you can find our point-by-point responses to the major comments and concerns of the reviewers and assigned academic editor.

- As required by **rev.#2** and by the **assigned academic editor,** we have better positioned the manuscript within the current related scientific debates, both by mentioning the emerging contributions about the so-called "infodemic", and by providing a more thorough clarification of our theoretical frame in addressing media (see section "Introduction"). In this respect, as rightly argued by **rev. #1**, although it is well recognized that the pace of newsmaking is quite different from that of scientific knowledge-making, for the purposes of our paper it is relevant that: ii) on one hand, media can be scrutinized as active agents contributing themselves to the development of the ways of managing the pandemic (i.e. media as performative agent; on this point see among others Cartwright 1998; Clarke 2009; Altheide, 2013); ii) on the other hand, media represent a valuable source of empirical data precisely for studying those processes. Following this theoretical frame, we clarified how in our study media are understood both as discursive arenas engaged in shaping public responses to the pandemic, and as a data source for analyzing how political institutions manage relations with scientific regulatory bodies and scientific communities for the sake of public health. Hence, in this perspective the concern is not so much to describe the ways in which media depict science and pandemic public policies; rather we focus our attention on how the concerned media can provide evidence about the reconfiguration of the nexus of science and politics during the early phases of the Covid-19 pandemic.

- As required by **rev.#1** and by the **assigned academic editor**, we provided a fully methodological rationale for our decision to focus exclusively on the so-called first pandemic wave (see lines 106-117). It is worth mentioning that we decided to focus on this timespan (January-June 2020) characterized by a relative lack of curative and preventive treatments for the Covid-19 disease, in which public health responses against the spread of the Sars-Cov-2 were primarily rooted in

lifestyle and behavioral changes, that is social norms and conventions, whose plausibility and legitimacy can be widely debated by mainstream media. Furthermore, it is crucial to clarify that the later timespan of the subsequent pandemic waves clearly shows a media landscape in which the nexus between science and politics is primarily centered around and dominated by the vaccination campaign debate. Hence, this aspect strongly hampers a thorough understanding of how the re-articulation of the scientific and political poles (in which the subsequent vaccination campaigns are rooted) actually occurred, and which effects it produced on the public governance of the health crisis;

- As required by **rev.#2**, in the section "Conclusion" we located more explicitly within the findings the notion of "politics' patronization" in relation to the "personalization of politics" in media accounts by further discussing how patronization refers to the key role of politics in defining the frame of reference for interfacing biomedical expertise and society, thus providing "its stamp of approval" to public health measures defined by major scientific and medical institutions. The findings allow us to argue that patronization unfolds by means of knowledge certification practices developed through specific political and institutional arrangements that allow certain public health measures to enter legitimately in the public sphere. As such, major political actors and political institutional arrangements neutralize individual scientific actors and the expert knowledge on which concerned public health measures are grounded. Hence such measures, by means of the intermediation of specific political regulatory tools, are brought back to the scientific institution itself, depicted as a neutral space for informing political decision-making over health crises. In this way, politics can impute its pandemic governance action to an alleged expert homogeneity and monovocality of science as an organic institution, thus eliding the fact that pandemic scientific discourses can actually evolve into pluricentric fields comprising a range of different experts and diverging positions over the same debated topic.

- As required by **rev.#3** and by the **assigned academic editor**, we fully describe how we address what can be defined as the demarcation problem between science and other thematic domains (see sections "Introduction" and "Data Analysis"), by clarifying both how we selected the articles in order to establish the corpora we analyzed by the means of topic-modelling techniques and how we build our classifier to discriminate the content of newspaper articles in terms of their scientific relevance. In doing so, we emphasize that we addressed the "demarcation problem" in an empirically-oriented manner. In this regard, even if our approach does not theoretically address the demarcation of science from other social activities (which is an issue quite far from the purposes of this study; see Gieryn, 1983), the article suggests that this problem is to be addressed in terms of a gradient of intersection of science with other fields rather than providing a clear distinction between well-bounded domains.

- As required by **rev.#2**, we included references to support the classification of the media as

progressive, moderate, conservative, neoliberal and Catholic, which is also a well-recognized set of categorizations used by political sociologists and political scientists (see line 209).

- ♦ As required by **rev.#2**, we described the pre-processing phase in the “Datasets” section, in particular by including details regarding the corpus creation, text preparation, stopwords, and the bag-of-words approach.
- ♦ As required by **rev.#2**, we clarified the issue of the three corpora as follows: in the “Materials and Methods” section we only mentioned the existence of several corpora, and we referred to the “Datasets” section for further details. In this latter section we outlined the steps for constructing each of the three corpora.
- ♦ As required by **rev.#2**, we added details about the Machine Learning (ML) methods we used. In the “Data Analysis” section we included initial candidate classification algorithms, the cross-validation procedure, the creation of training and test sets, and the manual labeling of those articles in order to extend the classification to our corpora through Supervised ML.
- ♦ As required by **rev.#2**, we pointed out in the “Introduction” that our topic modeling is based on the Latent Dirichlet Allocation (two sections earlier than in the original manuscript).
- ♦ As required by the **assigned academic editor**, and following the Plos One Policies, we made the “Sars-Cov-2 general corpus” data publicly available. Since the copy-rights of the article full texts are owned by several third-party organizations (i.e., the publishers of the eight newspapers), we provided all information necessary for interested researchers to gain access to the data: date, source (newspaper), and URL of each of the 58,646 articles belonging to the “SARS-CoV-2 general corpus.” This dataset is adequate to replicate our study findings, and it has been deposited in a public repository, Zenodo, under the following doi: 10.5281/zenodo.4624096 (Data from: Politics overwhelms science in the Covid-19 pandemic: evidence from the whole coverage of the Italian quality newspapers).
- ♦ The title has been modified by replacing “Sars-Cov-2 pandemic” with “Covid-19 pandemic”, which is actually more accurate for referring to the current global disease outbreak.

****

Once again, our grateful thanks for your care and attention. We hope that we have made good use of your efforts.

The authors
